# “It made me feel like a shit parent”: an intersectional analysis of pandemic mothering

**DOI:** 10.3389/fsoc.2024.1359827

**Published:** 2024-04-02

**Authors:** Holly Thorpe, Nikki Barrett, Mihi Joy Nemani, Grace O'Leary, Nida Ahmad

**Affiliations:** ^1^Te Huataki Waiora School of Health, University of Waikato, Hamilton, New Zealand; ^2^Independent Scholar, Denver, CO, United States

**Keywords:** motherhood, COVID-19, pandemic, intersectionality, Aotearoa New Zealand

## Abstract

The COVID-19 pandemic brought to the fore the everyday and exceptional challenges for mothers. Rarely, however, did research or social commentary acknowledge the multiplicities of motherhood during this prolonged period of risk, disruption, and uncertainty. This paper draws upon interviews with 24 mothers living in Aotearoa New Zealand during the pandemic, including women who were pregnant and gave birth during lockdowns, teenage mothers, single and low-income mothers, and working mothers. The sample was intentionally diverse, including Māori, Pacific, Asian and migrant mothers. Engaging an intersectional lens on motherhood and women's health, this paper builds upon and extends feminist research on mothers' experiences during the pandemic, highlighting the many different challenges facing mothers of diverse social, cultural, and economic positionalities and during various stages of motherhood. Across the sample, we reveal the significant emotional toll on mothers, particularly with the absence of critical social, medical and health support systems during lockdown periods and sustained social restrictions. Many of the women described how the pandemic affected their feelings about motherhood, prompting new reflections on their relationships with the home, family, work, and broader society. Despite some similarities, the pandemic experiences of Māori, Pacific, migrant and single mothers were further intensified by various forms of isolation, judgement, and discrimination. In this way, the pandemic shed light on the gendering of everyday maternal life, but also the need for more intersectional culturally and gender-responsive policies that acknowledge the multi-layered complexities of mothers' lives.

## Introduction

The emotional, social, physical, and financial challenges for mothers were brought to the forefront during the COVID-19 pandemic. Rarely, however, did research or social commentary acknowledge the multiplicities of motherhood during this prolonged period of disruption and uncertainty. Mothers are not a homogenous group and their experiences before, during and beyond the pandemic are highly variable based on an array of intersecting considerations, including familial and social support structures, number and age of children, work responsibilities, and family financial security. Furthermore, wider social systems of inequity and injustice (i.e., colonization, racism, sexism) impact mothers in very different ways. In this paper we engage an intersectional feminist lens to highlight the many different pandemic-related challenges facing mothers of diverse social, cultural, and economic positionalities and during various stages of motherhood.

Our analysis draws upon interviews with 24 mothers living across Aotearoa New Zealand (hereafter Aotearoa, the Indigenous name for New Zealand) during the pandemic, including women who were pregnant and gave birth during lockdowns, teenage mothers, single and low-income mothers, and working mothers. Across the diverse sample, we reveal the significant emotional toll on mothers, particularly with the absence of critical social, medical and health support systems during lockdown periods and sustained social restrictions. Many of the women described how the pandemic affected their feelings about motherhood, prompting new reflections on their relationships with the home, family, work, and broader society. In this way, the pandemic shed light on the gendering of everyday maternal life, and the need for more gender-responsive policies that acknowledge the multi-layered complexities of mothers' lives. This paper builds upon and extends intersectional research on motherhood and women's health, and a growing body of feminist research focused on mothers' experiences during the pandemic.

## Literature and conceptual framing

During the pandemic, scholars across an array of disciplines set out to understand the impacts of this radical social disruption on mothers' health and wellbeing. In so doing, this research built on and extended decades of feminist scholarship on families and motherhood (Rich, 1986). The anthology *Mothers, Mothering and COVID-19*, edited by Green and O'Reilly (2021) is an important contribution to this literature, documenting how the social, financial, emotional and health challenges and concerns impacted mothers in wide ranging ways. While some have revealed the positive aspects of the pandemic for middle- and upper-class mothers Somogyi et al. (2022), most feminist research on pandemic mothering has highlighted how gender inequities were exacerbated during this period, negatively impacting women's health and wellbeing. Various scholars have taken up and extended the concept of intensive motherhood to understand how pre-existing demands on mothers' time and energy were further amplified, or ‘intensified', during the pandemic as many mothers carried the majority of childcare during lockdowns, often while also managing their jobs and other community and family care work (Cummins and Brannon, 2022; Limonic, 2023). Prior to the pandemic, many mothers felt the burden of “unrealistic and overwhelming conditions of motherhood”, but during COVID-19 many mothers experienced increased feelings of failure amidst “good” mother myths in the context of the “paradoxical freedoms” under pandemic time (Friedman et al., 2021, p. 47). With mothers carrying heightened levels of care work (i.e., homeschooling) and new pressures of managing their paid labor from within (or outside) the home, they have “little to no respite from their 24/7 schedule”, adding “income or employment loss, financial or housing instability, food insecurity, single parenting, abusive situations, or recent experiences of migration” such that the “stress is amplified” (O'Reilly, 2020, p. 1).

Researchers have focused on particular phases of motherhood, including the pressures on new mothers during the pandemic. Research has focused on increased challenges regarding mental health (Kinser et al., 2021) including postnatal depression and anxiety (Ryan and Barber, 2022), and emotional wellbeing (McLeish et al., 2022) experienced by pregnant and new mothers during the pandemic. Drawing upon data from a National Maternity Survey conducted in the United Kingdom during 2020, McLeish et al. (2022) identified three key themes, (i) Chaos: impact of uncertainty, (ii) Abandoned: impact of reduction in care, and (iii) Alone: impact of loss of social support. Although the mothers valued the maternity care received during the early stages of the pandemic, many experienced additional stresses from “chaotic changes and reduction in care during the pandemic”, from “health professionals' own uncertainty and anxiety”, and also from “restrictions on essential social support during pregnancy, labor and birth” (McLeish et al., 2022, p. 868). Others focused on the challenges experienced by various groups of mothers, including refugee mothers (Adamson, 2023), mothers of children with disabilities (Currie et al., 2023; Good and McBride-Henry, 2023) and low-income single mothers (Radey et al., 2022). Such groups were often required to perform heightened levels of care work with less social, emotional, and financial support.

Another key focus in the pandemic motherhood literature is on working mothers. Often drawing upon feminist theorizing of work and labor, scholars have highlighted how the pandemic exacerbated uneven, gendered distribution of domestic and familial care work (Martucci, 2023), with working mothers often expected to pick up additional responsibilities (i.e., home schooling children, caring for elderly parents) while maintaining their own professional careers or reducing their working hours. For many mothers, the pandemic brought to the fore the pressures of “good” motherhood, with many experiencing feelings of failure, guilt and/or exhaustion, negatively impacting their own mental health and wellbeing (Whiley et al., 2021).

### Intersectional motherhood

In this paper we build upon and extend research on motherhood during pandemic times by drawing upon the Intersectionality Framework (Crenshaw, 1991). Intersectionality was originally conceived as an approach to articulate the experiences of oppression faced by Black women within the legal system in the United States. Intersectionality proposed that although Black women face discrimination for positionalities shared with other groups (e.g., White women, Black men), they also experience intersecting forms of oppression, including racism, sexism and classism (Crenshaw, 1989; Collins, 2000). Feminist scholars from a range of disciplines and working across many geographical, social, and cultural contexts have taken up and applied the concept of intersectionality, including research on families and motherhood (Anaya, 2011; O'Hagan, 2018). In so doing, researchers have examined the experiences of pregnancy and motherhood among those “located at the intersecting axes of disadvantage”, revealing the powerful “influence of stigma and the acknowledgment of race, class and gender subordination as interlocking forms of oppression” (Mantovani and Thomas, 2014, p. 45). In a recent critique of research on “intensive mothering”, Minnotte (2023) calls for more intersectional approaches that recognize that “enactments of motherhood are varied, forming a mosaic of motherhood enactments informed by mothers' social locations, including their positions in racialized and classed hierarchies” (p. 1).

During the early phases of the COVID-19 pandemic, Crenshaw (2020) applied her framework, highlighting “the intersectional vulnerabilities that COVID lays bare” (n. p). Many others have also applied intersectional approaches to highlight how the pandemic heightened risks to those with “multiple intersectional burdens”, calling for more targeted support to the most disadvantaged groups (Moen et al., 2020; Ho and Maddrell, 2021, p. 1). Feminist scholars have argued for the importance of intersectional approaches to deepen understanding of how pre-pandemic inequities and injustices associated with race and gender coincided with the pandemic to impact women's physical and mental health risks, their economic and social security, their experiences in the home, in public spaces, and the workplace (for example, Baig, 2021; Fulcher et al., 2023).

Some feminist scholars have argued that intersectional approaches are also necessary for understanding mothers' diverse experiences during and beyond pandemic times. For example, Hillier and Greig (2020) advanced a gendered intersectional analysis of caregiving among mothers in Canada. In the context of the “double pandemic” of COVID-19 and the Black Lives Matter movement in the US, Hassoun Ayoub et al. (2023) revealed how systemic inequities embedded in many institutions (e.g., healthcare, law, education) exacerbated Black mothers experiences of worry, anxiety, stress, and fear. Similarly, McKinney and Meinersmann (2022) make the case for more intersectional approaches to examine mental health among Mothers of Color (MOC):

Although the demands of motherhood alone may contribute to adverse mental health conditions, the intersection of this role with other socio-cultural identities (e.g., race, ethnicity, gender identity, sexual orientation, socioeconomic status) elicits unique circumstances that scholars must consider (p. 598).

In the context of Aotearoa New Zealand, Martin-Anatias et al. (2021) reveal the “intersectional specificity” of pandemic mothering, focusing on the experiences of Indonesian migrant mothers. While this group of mothers reported enhanced relationships with their children and partners during lockdown periods, they also experienced feelings of “anxiety, frustrations and inadequacy with homeschooling their children in English” (p. 9).

Most of the intersectional research on pandemic mothering has focused on particular groups of mothers (i.e., Black mothers, Indonesian migrant mothers), and in so doing rich details have been provided into these mothers' specific challenges. In this paper we extend existing research with a sample of mothers from diverse social, cultural, and economic backgrounds. Drawing from a larger multi-phased project focused on New Zealand women's experiences of wellbeing during the pandemic, we highlight the value of intersectional approaches that recognize the multiplicities of motherhood, and how the highly variable social, cultural, and economic conditions of mothers' lives impact their experiences during and beyond the pandemic. In so doing, we also build upon and extend a small but growing body of literature that has adopted intersectional approaches to understand the pandemic experiences of women in Aotearoa New Zealand (Thorpe et al., 2023b), including young women (Thorpe et al., 2023a), migrant women (Soltani and Thorpe, 2023), and frontline health workers (Sumihira, 2020).

## Context and methods

Responding to the first domestic cases of COVID-19 in March 2020, the New Zealand government issued a full national “lockdown” for at least 4 weeks. The borders were closed indefinitely except for returning national citizens. The government adopted a science-informed approach and a strong communication campaign, and as a result, was temporarily successful in its elimination strategy. Then in mid-late 2021, the arrival of the Delta, and then Omicron, variants prompted additional national and regional lockdowns (i.e., 107 days in Auckland; 65 days in the Waikato) to control outbreaks, limit interregional spread, and allow time for the vaccination program to come into effect. National and regional lockdown periods were strictly enforced, with the government implementing some of the world's most rigorous and stringent efforts to minimize the spread of the disease and mass casualties. While this strong governmental response saved an estimated 20,000 thousand lives, as the pandemic wore on, it faced heavy criticism from both inside and outside the country (Baker et al., 2023). By 2022, Aotearoa was reopening borders, removing vaccine mandates, and largely putting the responsibility back on citizens to navigate the risks individually. Despite a government strategy that initially sought to protect the most vulnerable, the pandemic exposed existing social issues and injustices (i.e., poverty, a housing crisis, health disparities). The negative effects of COVID-19 were felt disproportionately by ethnic minorities, particularly Māori and Pacific communities who experienced higher infection and fatality rates due to an array of intersecting variables, including underlying health conditions, socioeconomic disadvantage, and structural racism in the health system (Steyn et al., 2021).

With ethical approval from the University of Waikato, we conducted interviews with a total of 17 mothers aged 24–43, and one focus group with 7 young mothers aged 16–19. The latter were in the same school programme designed for young mothers, and they expressed a preference to participate in a focus group, which we accommodated willingly and within the permits of our ethical approval. All participants had the option of either full anonymity or partial identification (i.e., age, ethnicity, number of children). Each member of the research team contributed to the data gathering, reaching out to women in their social, work, and familial networks to invite participation. All who expressed an interest in participating were provided with an Information Sheet that detailed their ethical rights as participants, and all participants completed an online or hard-copy Informed Consent form. The women ranged considerably in the social and economic circumstances of their lives, with our sample including pregnant women (2) and teenage mothers (7), as well as single mothers, and working mothers, and some combination of these positionalities (e.g., teenage single mothers, single working mothers). The women had varying caring responsibilities, including one (10), two (10), three (1) and four (1) of their own children at home, with some also caring for other children (i.e., younger siblings, cousins, nieces, and nephews) or vulnerable family members (i.e., elderly parents or grandparents). They lived in urban, rural, and semi-rural locations across the country, but with the majority residing in the North Island. A few mothers were caring for children with serious health conditions, or themselves were living with chronic health issues. All self-identified as women, and while most referred to heterosexual relationships (i.e., husband, partner, or ex-partner) during the interviews, we did not ask participants to specify their sexual identities or relationship status. Our sample included mothers of diverse cultural and ethnic identities, including Pākehā (NZ European) (3), Māori (Indigenous to Aotearoa) (3), mixed ethnicity (i.e., Māori-Samoan, Māori-Pākehā, Cook Island-Māori) (9), immigrants (Peruvian, Chilean, Indian, South African, Canadian, English, Iranian) (7) or with lineage to a specific Pacific nation (i.e., Samoan) (2). The social and cultural diversity of our sample was facilitated by the personal and professional networks of our research team who identify as cis-gender New Zealand born Pākehā, Māori, Māori-Samoan, Māori-Pākehā, and an American Muslim woman. Wherever possible, we matched researchers with mothers of the same ethnicity.

The interviews (and one focus group) were conducted over an 18-month period, with half the total number of participants conducted in 2021 (12) and 2022 (12) due to convenience and funding support. While most of the interviews took place in person in spaces of convenience to the mothers (i.e., in their home), some occurred digitally using Zoom. The focus group took place in a private room at the school attended by the young mothers. The interviews ranged in length (from 50 min to 1.5 h) based on the availability and energy of participants, with each digitally recorded and then professionally transcribed. The same guide was used for the interviews and focus group, and included 22 questions organized into five sections designed to explore how the pandemic (and particularly lockdown periods of 2020 and 2021) impacted the mothers' overall wellbeing, their experiences in the home, school, work, their relationships with friends and family, engagement with social media and digital technologies, and activities and practices they used to support their own and others wellbeing during the pandemic. The focus group included young mothers who knew each other well, and thus there was rich discussions between participants. Due to the in-group dialogue, the focus group required greater flexibility in how the interview guide was used. The individual interviews offered more personal information and, at times, greater depth and nuance. Wherever possible, we worked to ensure consistency in the questions and prompts for interviews and focus group. Adopting a semi-structured approach, we sought to create an environment where the mothers felt comfortable to respond and (re)direct the conversation such that they could speak to topics of importance to them. The timing between the two periods of data collection did not reveal any significant differences. While mothers interviewed during 2021 where temporally closer to the lockdowns, with some prompting, all mothers spoke vividly of their experiences during pandemic lockdowns.

Each interviewer took care to explore how culture, ethnicity, household dynamics, living circumstances (i.e., rural, semi-rural, urban), and socio-economic conditions impacted the mothers' pandemic experiences. Incorporating intersectionality into the research design (Windsong, 2018) also required us to reflect regularly on how our own multiple and intersecting identities were shaping our relations with participants during interviews and analysis. Two members of the research team are mothers with young children, and across the authorial team we reflected upon how our varied relationships with motherhood and understandings of family shaped our access and interpretations of the data. We reflexively acknowledged both similarities and differences with our participants, while always working to practice a feminist ethic of care (for participants and each other) throughout the project (Jeffrey and Thorpe, 2023). Drawing upon our cultural knowledge, we carefully navigated our researcher positionalities, and took care to respect participants' cultural and religious practices. As per Māori and Pacific cultural practices, kai (food) was provided in each interview and focus group, and where appropriate, karakia (Māori or Pasifika cultural and/or religious prayer) were offered as a blessing over the kai and/or the meeting. All participants were given a gift voucher (koha) as an acknowledgment of their time and expertise.

## Analysis

In this paper we engaged Braun and Clarke's (2021) reflexive thematic analysis to identify key themes. We began by analyzing the transcripts individually, and then brought the transcripts into dialogue with feminist literature on motherhood and intersectional research on women's health during the pandemic. Drawing upon our individual and collective feminist perspectives, familial and cultural knowledge was an important part of our intersectional and reflexive analysis process (Landry, 2007; Christensen and Jensen, 2012), particularly in the latter stages as we discussed the various ways culture, ethnicity, gender and living circumstances were intersecting to impact mother's lives differently during the pandemic. Through this multi-phased process of analysis and collaborative writing, we came to identify the unique challenges for mothers of different positionalities, as well as examine common themes across all mothers.

### The multiplicities of pandemic motherhood

This section consists of three parts, with each focusing on the experiences of different groups of mothers (i) those pregnant, giving birth and caring for newborn babies during the pandemic, (ii) single and low-income mothers, and (iii) working mothers. Then in the following section we will explore high level themes across these groups of mothers.

#### “You can't get that time back”: pregnancy, birthing and newborn babies

Some of the mothers in our sample were pregnant with their first or second baby during the various New Zealand lockdowns. During the interviews, these mothers described experiencing various health concerns, including a fear of catching COVID-19, and concerns about lack of familial support during the birthing process:

I was worrying about getting it, what would happen to the fetus? Then when you actually go into birth, when there was an outbreak, people weren't allowed to have their partners in the delivery room, so we didn't know what was going to happen. The thought of [partner] not being there really freaked me out. Just having that in the back of your mind and worrying about whether your new-born would have some kind of health issue and then COVID would come. There were all those sorts of things that added that extra layer of anxiety on top of all the things you already worry about being pregnant (Emilla, first pregnancy).

Pregnant women were navigating many different sources of information to try to understand the risks of COVID-19 while pregnant. During the early stages of the pandemic, scientific information was emerging very quickly, and there were long periods of time with much uncertainty due to the quickly evolving nature of the virus and its effects on human health. This context caused considerable stress and anxiety for pregnant women and new mothers who felt an “overwhelming sense of responsibility” to their (unborn) babies (Gray and Barnett, 2022).

The lack of services during this time caused many of the mothers, particularly those pregnant or with newborns, to experience various forms of uncertainty, stress, and anxiety. Many described logistical challenges in meeting their midwives or other medical professionals due to social isolation restrictions in place:

It was a really anxious time because none of the services were available. I was trying to ring the numbers and there was nothing there, no support. It got up until I was throwing up every day and I knew I was pregnant and the hormones were high. I went to a scan on my own, because [husband] wasn't allowed to come with me (Beth).

Some experienced challenges with accessing social and emotional support. For example, one of the mothers experienced a miscarriage during the first nationwide lockdown, and recalls this as a particularly traumatic period of her life:

The nurse called me up and said, “You've got to go to ED now because you're still bleeding.” I went there, they dropped me off and she said, “You've got to be prepared for a D&C [dilation and curettage]”. So I went there. Going to the hospital was traumatic. They were very busy, I didn't get any emotional support. I was on my own and it was painful and awful. I came home, recovered. Then weeks after that, I had quite an emotional breakdown, I think because of the events.

Some of the mothers expressed sadness and frustration by COVID-19 rules that restricted family support during special moments in their pregnancy journeys, particularly scans and birthing:

You can't get that time back. For me personally, I'll never get to experience my first birth with my mum there. Or my scans. I had to go on my own, my family waited outside in the carpark. (Shanti, teen mother)

Attending medical appointments alone, without family support, was upsetting for some mothers (particularly teenage mothers), whereas others accepted such conditions as part of a national campaign to limit the spread of the virus.

In the context of COVID-19, vaccinations were highly politicized “biomedical technologies” playing a significant role in the construction of the fetal and maternal body, and discourses of risk and responsibility (Lupton, 2012, 2022, 329). While the mothers in our study had widely ranging views on vaccination, many experienced various forms of judgement, misinformation, and miscommunication from the public and medical health professionals. For example, first time migrant mother Keeja recalled her distress upon receiving conflicting information about vaccination:

The same day I did my home pregnancy test and it was positive was the same day I did my first dose of vaccination, because I had already booked it. They tell you to let them know and I said, “Well, I just found out today that I'm pregnant.” They were like, “Oh, do you want to get vaccinated?” I was like, of course, I can't believe you're asking me this!

For many mothers, vaccination and masking were important measures to protect themselves and their babies, while others chose not to be vaccinated in fear of side-effects on their babies. With significant social restrictions based on vaccination status, some unvaccinated mothers described feeling angry about missed opportunities for their families to join them at medical appointments (i.e., scans, physiotherapy) and important milestones in their journey into motherhood:

I didn't get vaccinated while hapu (pregnant)… because I don't know how that's going to affect my baby yet. But I would be in so much pain, my midwife would recommend me to go get massages, and chiropractic stuff, and I had to sit at home in pain because of the vaccine rules. (Avi, teen mother)

I didn't like how you had to be vaccinated to go to things, like even scans. My baby daddy didn't come to my scans because everyone had to be vaccinated. (Hotu, teen mother)

For these young Māori mothers, recalling their pregnancies during lockdowns and vaccination restrictions, evoked strong feelings of loss, grief and even anger toward the government. Herein our findings align with Thornton and Reich's (2022) research on Black mothers and vaccine refusal in the US, highlighting how structural gendered racism and concerns about state intervention shaped vaccine decisions.

Although the women had different views on vaccination, many felt that their competency as a ‘caring and responsible' mother was being judged by strangers, family members and medical professionals based on their willingness to be vaccinated or not, and their efforts to minimize risk:

I had a lot of medical issues with my pregnancy, and I'd go to hospital a lot. And it's just that constant badgering or the sly, “oh, you're already not going to be a good mum, because you're not getting vaccinated”, “Think about your child”, “do what's best for them”. What happened to, ‘my body, my choice'? I felt discriminated against because I wasn't vaccinated. (Shanti, teen mother)

I remember one lady was in the lift with me and she turned to me and said, “Oh, I hope you're being careful.” I was like, “yeah, because I'm fucking going around [NZ city] trying to catch COVID. I want to get my baby sick [sarcasm]”. You become like public property when you're pregnant, but those sorts of comments were a whole other thing. (Emilia)

Outside of pandemic times, pregnant women and mothers of newborns experience high levels of “regulation, monitoring and control”, as well as social judgement in their approaches to mothering and the decisions they make to care for their babies and themselves (Longhurst, 1999; Lupton, 2012, 329). During the first two years of the pandemic, the mothers described heightened levels of judgement, particularly as pregnant women and new mothers navigated unknown risks of COVID infection and vaccination, often without the medical and social support required to make informed decisions. In this way, our findings align with Manca's (2021) research on pregnant women's experiences of risk and intersectional power relations during the early stages of the pandemic. According to Manca (2021), medical responses to COVID-19 were “(dis)organized within pre-existing economic, racial, colonial, and patriarchal power relations that disadvantaged some pregnant women more than others” (321). As our research shows, moral and medical judgements were indeed highly racialized, with young Māori, Pacific and migrant mothers disproportionately facing the brunt of health professionals questioning and scrutiny.

According to a recent report, the “single largest cause of maternal death in Aotearoa is suicide”, particularly affecting wāhine Māori mothers (Hoki, 2019). With the pandemic further amplifying mental health issues, and with the absence of many structures of formal and informal support, intersectional approaches that recognize the compounding factors of ethnicity and socio-economic deprivation (as a result of colonization, and ongoing racism in everyday life and health and social service provisions) on mothers' health and wellbeing are necessary to provide the culturally-responsive care and support for those most impacted (Barrett et al., 2023).

#### Single and low-income mothers

Our sample included several single and low-income mothers for whom the pandemic posed many emotional, social, and financial challenges. Recent research (Mako Mama - Mangopare, the Single Parents Project) conducted with over 3,500 sole parents in Aotearoa has documented the various forms of stigma and discrimination they experience (i.e., housing, welfare, social agencies) (Domett et al., 2023). There is a distinctly racial element to such discrimination. According to New Zealand's Ministry of Social Development, 46 percent of sole parents on a benefit (welfare) identify as Māori, 28 percent as NZ European, and 12 percent are of Pacific Island heritage (Ministry of Social Development, 2021). Most of the single and low-income mothers in our study were of Māori and/or Pacific ethnicities, many of whom have experienced various forms of racism throughout their lives (i.e., schooling, employment, health systems), while the maternal and welfare services they receive position them as subordinates (Gray and Crighton-Hill, 2019). According to Adcock et al. (2019), young Māori mothers are subject to “Eurocentric medical, disciplinary, and colonial gazes—through exclusionary health, education, and social services, and public prejudices—that see them as abnormal and in need of regulation” (250). However, their research also showed that, with support from their whānau (families), many young Māori mothers are “working hard to provide a positive future for themselves and their children” (250). With whānau often playing such an important role in the support networks for single and low-income mothers (and particularly Māori and Pacific wāhine/women), extended lockdowns caused considerable distress when access to essential family and child-care support was no longer available or highly limited.

Of particular concern for these mothers was the loss of child-care (i.e., school, daycare, kindergarten) and family support (i.e., grandparents, sisters). Without any reprieve from their full-time childcare responsibilities, the mothers described the significant impact on both them and their children. For example, Mila, a single mother with two young children, recalls:

The boys started getting restless, and just not being able to get out and about was doing my head in, plus having the boys here with myself for almost three months now was driving me crazy. It was hard because they were playing up because they wanted to go see Nanny and Poppa, they couldn't understand why they couldn't go to school, see their friends, or play outside with the other kids like they usually do. Seeing how tough it was for them, it was hard on me, and it just made me feel like a shit parent really.

For many single low-income mothers, getting access to food and supplies during lockdowns was another significant challenge. For some, access to public transport (i.e., buses) or childcare made supermarket visits difficult. For others, particularly those with children with allergies or nutritional needs, access to affordable supplies required highly creative approaches:

I couldn't find anything [toddler] was able to eat because he is very lactose intolerant. I'm only on a benefit so I was always going for the dollar packets of pasta and baby wipes, but all the cheaper items were always run out. Me and three of my siblings would go to the supermarket… each person had a list of items to get… and it will take a whole day just to get our regular shopping. Also, getting clothes was hard because the boys were out growing things so fast. (Narla)

Continuing, Narla recalled the challenges of being a single parent while she and her children (both under 5 years) had COVID-19. Unwell and without any energy, she was unable to feed, clothe or change them:

I would only have 15 min of energy and in that 15 min I would open as many snacks as I can for the boys to help themselves throughout the whole day. Then I'd fall asleep. I'd pass out. Dinner time I would just Uber Eats a really big meal and they would just come and help themselves. I would fall asleep. It was horrible. I'm surprised I survived that whole thing on my own. I was mentally unstable.

As the quotes powerfully illustrate, the pandemic took a particularly strong toll on the emotional, social, and physical health and wellbeing of single low-income mothers and their children. Our findings here align with research in the US that showed financial instability significantly exacerbated the mental health impacts (anxiety, depression, loneliness, and stress) among Black mothers, with stable income serving as “a protective factor for anxiety” (Ibekwe-Okafor et al., 2023, p. 694).

A key theme for the single low-income mothers was the importance of informal social support networks. Some mothers were grateful to their family members who went to great lengths to support them from afar. For example, Lady recalled: “Mum was calling and talking to the kids every day. And that made things a lot easier”. Others described family, neighbors, and even the police, stepping in to relieve the pressure at critical moments:

I'd be at home by myself with my sisters, who are nine-year old twins, and then my daughter who was a newborn. But my auntie, she lives down the road, so she was always coming to see me, hang out, help me clean the house with the kids, because they didn't listen to me. (Porsche)

Recognizing the seriousness of the situation, some friends and family broke protocol to support the single mothers. For example, Mila described the critical roles played by a neighbor and her friends, while she struggled with mental health issues during the 107-day Auckland lockdown:

I had my neighbor, she shares a wall with me. …if she could hear me screaming at the boys and the doors are closed and she can hear me through the walls, she'll just let herself in and deal with the boys while I calm down and take 10 min to myself.

My friends are awesome. I called them up and I was like, “Look, I'm not doing too good.” Then they all came over. They were like, “stuff the lockdown”. And they all showed up. A few hours with my friends … just keeps me going.

For other single mothers, it was the financial aspects of the pandemic that posed the biggest challenges, with family members supporting them between jobs:

Mum and dad were my biggest supporters. When I dropped down to part time [due to redundancies at work], they offered to cover [daughter's] school expenses. As well as my brother, he ended up helping out with the grocery shopping. (Alyssa)

Our findings align with those of Radey et al. (2022) who identify the importance of formal and informal networks for exchanges of support for low-income mothers during the pandemic.

Yet not all single mothers found support among their family and friends. Some described how the intensity and stress of the pandemic brought to the fore existing and new issues in their relationships with family members. For example, Narla described feeling disappointed when her parents, sisters and friends failed to understand the difficulties she was experiencing, and were unable or unwilling to look after her two young children:

COVID has turned our world upside down in a bad way, in a very, very bad way! My friends with kids don't get it because they've all got partners to help out with the kids and stuff. And my family are too tired from looking after my nephew 24/7 that when I ask for help looking after the kids, they just don't want to. My sister and I used to talk every day, but now we don't talk or see each other anymore.

After many weeks of being the sole caregiver for two young children, and without any family support, Narla felt abandoned and alone (McLeish et al., 2022).

Another mother of two young children recalled how the intensities of pandemic life surfaced challenges in her relationship with her partner, with instances of domestic violence that prompted her to leave the family home:

Our relationship wasn't fabulous anyway, but I think just being locked down just the four of us for that length of time just really brought out the bad sides. I had to make that decision, not only for myself, but for the kids because it was just such a toxic environment for them to grow up in. And the domestic violence happened in front of my oldest… I just had to make that call. I became a solo mother. I had to become a whole lot more independent, emotionally and financially. And yeah, we moved back in with my parents for 6 weeks before I found my own place. (Lady)

As Broughton et al. (2022) have argued, mothering in the context of intimate partner violence requires feminist intersectional approaches that move beyond dominant ideologies of “normative motherhood, deficit and grit/resilience”, and toward approaches that emphasize “strengths and equity” (p. 3974). Such approaches become even more important in the context of the pandemic, where cases of domestic violence and intimate partner violence grew exponentially, and support systems available for women and their children were heavily reduced (Usher et al., 2023). In sum, single low-income mothers' relationships with existing networks, and particularly family and friends, were either “strengthened or dissolved” in the context of COVID-19 (Radey et al., 2022), greatly impacting their capacity for coping during this exceptionally difficult period.

#### Working mothers

Mirroring international literature, the working mothers in our sample overwhelmingly spoke of the challenges of “juggling” paid word and childcare (Whiley et al., 2021; Martucci, 2023). Spreading their energy, time and attention across their personal and professional duties took a toll on mothers' health and wellbeing, and in some cases, that of their wider families:

The kids needed to get out. They would just become hungry for your attention and so having to deal with that stuff plus work stuff, plus often [husband] was being called away as an essential worker, I'd have days at home where I was just stuck in this place of trying to juggle too many things. I think that was probably the hardest thing, because so many people were depending on me through work. I would have an internal battle, do I half-arse watch the kids doing something and try and deal with some work stuff at the same time, or do I ignore the work stuff and just deal with the kids, or ignore the kids and deal with the work stuff? (Karen)

Mentally it's tough. It has also hugely changed how I parent.… It has taken its toll on my patience and tolerance. It sounds really awful, but even to a certain extent, the enjoyment because there's so much more expected of you to do outside of the parenting stuff, that it takes some of the parenting enjoyment away. At the end of the day, when we finish the day of homeschooling with kids, then you don't necessarily have the same burning desire to go out and kick a soccer ball round with them anymore. (Shelley)

For many working mothers, the pandemic surfaced feelings of exhaustion, guilt, resentment, and even anger, further complicating their interpretations and experiences of “good motherhood” (Whiley et al., 2021).

From our sample, mothers in more demanding jobs (i.e., leadership and management roles), those with toddlers, and/or with partners in essential worker roles, experienced heightened challenges in navigating the demands of work and motherhood:

Trying to manage two full time jobs plus caring for a toddler… I felt really, really burnt out… It's just this constant grind. It was just this constant feeling of I'm not doing anything well, I'm just drowning in life, basically. (Emilia)

While some mothers (and those in mothering roles) struggled to manage the dual responsibilities of childcare and work, others found creative strategies to entertain young children while they worked:

What was really difficult was trying to teach [online] and asking the 2 year old not to be a 2 year old. We had this game we'd call “Anaya's time on the fala” (Samoan woven mat). So, if I was teaching, whatever item we put on the fala would be her toy to play with… while I was doing my classes. Usually she'd have the chalkboard or she'd have a book or she had some play dough or paints, whatever it was on the fala, and she couldn't go anywhere but on the fala. It helped me manage where she was, and I could get my stuff done (Sage).

This creative example of multi-tasking (online teaching while keeping a toddler entertained) highlights the importance of intersectional approaches that reject dominant framings of “intensive mothering” and recognize culturally-specific enactments of mothering and care work (Minnotte, 2023). Across our sample, however, the types of work the women were doing from home, and the levels of support within the family (i.e., partner at home; extended family living in the same household), significantly influenced mothers' feelings of competence in managing their multiple roles, and thus emotional wellbeing during the pandemic.

Many of the working mothers demonstrated a heightened awareness of the uneven gendered distribution of childcare and domestic labor during lockdown periods:

It was chaotic, to be honest. … [my husband] and I, we share responsibilities but at the end, I think it's obvious that the woman has to do more of the job, especially when the kids are small. (Shelia)

We had gender-based division of labor in my house, but when the kids needed something, they came to me. So, [husband's name] doesn't get 17 interruptions in a day. I do! The work-from-home impacted women more, there's an element of feminism still! (Powm)

Recognizing the impact of such inequalities, some parents worked to find more equitable distributions of domestic and emotional labor. For example, with both Emilia and her husband working full-time and caring for a toddler, they “tried to deal with it by splitting up the day” [of childcare] between them.

As well as observing gendered dynamics within the home, some of the working mothers identified highly variable approaches held by their employers. Some mothers spoke positively of their organizations efforts to develop gender-responsive policies and practices that recognized the challenges for working mothers during the pandemic. For example, Karen felt strongly that there had been a widespread social change in employers understanding and empathy for working parents, with a focus more on worker wellbeing:

I think that first lockdown where suddenly society gave you permission—well, certainly my employer gave us permission to focus on your family and they kept asking questions, “what are you doing to look after yourself?” It really shifted this culture into this place of “it's okay if, in order to keep your kids well, you need to go for a bike ride in the afternoon, or a walk, or some meditation”. … You suddenly became very cognizant of these parents who were stuck at home with children trying to hold down a 40-h week, very limited IT at home, no quiet space to work. This big shift started to happen where taking time out to do things to keep yourself well became okay.

Yet other mothers experienced workplaces that failed to acknowledge the additional demands on their time and energy. For example, Emilia quit a job where her employer failed to consider the difficulties for working mothers during lockdown:

I think it's made me realize the importance of work/life balance, that's why I changed jobs as well. I didn't feel like I had an employer that was very understanding during that time. They still expected that I'd be producing the same amount of work even though I had childcare responsibilities, and we were trapped in this house. They didn't respect my hours and stuff that I'd asked for. I feel like I've moved to a more family friendly employer now.

Herein our findings support international literature that has revealed the gender-responsive approaches by workplaces to support working mothers are so important, but highly uneven (Brewer et al., 2023).

### Commonalities: child and mother wellbeing

The previous section adopted an intersectional lens to highlight the multiplicities of mothers' pandemic experiences. In the second part of the paper, we identify three themes common across our diverse sample of mothers, particularly (i) concerns for their children's health and wellbeing, (ii) the impacts of the pandemic on mothers' health and wellbeing, and (iii) the various strategies mothers employed in their everyday lives to support themselves and other mothers.

#### Concerns for children's health and wellbeing

All the mothers expressed their concerns for the physical health of their families, particularly the uncertainties of how COVID-19 would impact their children. For example, Emilia explained: “My top concerns are if [baby name] got sick and reacted really badly to it. I'm worried about that!” Such concerns were heightened among mothers of children with health conditions diagnosed before, during or after COVID-19:

My daughter has bronchitis, a respiratory condition that she's hospitalized for a couple of times. I was quite concerned about her coming into the pandemic, so that was quite stressful. (Lady)

He [asthmatic son] was having seizures for over a minute. It took over 20 min for the ambulance to come. Before the COVID wave hit, it would take <10 min for an ambulance to come. Because of COVID, they needed the beds. So, it was doctor visits every single day while [his] obs and stats were still up. The doctor suspects that his lungs have damage from having COVID. I was told if he was ever to get COVID again, this trip might be the one that would be a major life or death. (Narla)

As highlighted in the comments from Narla, the compromised health systems (i.e., ambulance arrival times, availability of hospital beds) were a grave concern for mothers of children with health conditions. Adopting an intersectional lens, however, it is important to acknowledge that Māori and Pacific children are disproportionately affected by upper respiratory tract infections, asthma and chest infections, primarily due to inequitable access to warm and dry housing (BPAC, 2008). They also experience greater barriers in accessing medical care (Jamieson and Koopu, 2007). Thus, the heightened emotional labor of Māori and Pacific mothers' worries for their children's health is shaped by the ongoing effects of colonization, and casual and institutional racism in the Aotearoa health system.

Some mothers also worried about sending their children to daycare, kindergarten, and school, and described intensive thought processes, weighing up the risks and rewards of attending: “What's the risk factor? How risky is it for kids to go to school? How risky is it for kids to go to kindy? It's just a daily negotiation of risk” (Aisha). Importantly, such considerations often required mothers doing a lot of their own research amidst a context of competing information, as well as misinformation. A few mothers also discussed new complex considerations and parental responsibilities, pressures, and challenges, of keeping sick children at home:

Now that we're back in a phase where the kids are at school, I constantly worry, should I be sending them to school? If they get a snotty nose and I'm thinking about sending my kid to kindy because they're fine and I've got work to do, but then I think: “oh my gosh, am I the worst mother in the world?” If my kids are sick, I don't want them making other people sick, but I've got all this [work] to do! (Shelley)

Worrying about children's health, and the new responsibilities on parents to make ethical decisions that do not expose others to risk, are examples of the heightened stress, and the emotional load carried (predominantly) by mothers during the pandemic.

The mothers also worried about the impact of the pandemic on their children's socialization and mental health and wellbeing. Some referred to their “pandemic babies” who had spent many months at home with very little interaction with others:

I had my baby in the big lock down. We weren't allowed to go anywhere at all. My daughter didn't get out until she was about 6 months old, and that was for a powhiri (Māori welcoming ceremony). She [baby] was like this [eyes widened], and she just looked around and started crying (Avi, teen mother)

Other mothers witnessed changes in their children due to extended lockdowns, and worried for their mental health and wellbeing because of isolation from friends and disruption of the rhythms and routines of school:

He was crying because he felt lonely. He hates COVID and he just doesn't want to be here anymore because he's lonely. He can't see friends, he can't go to school, he loves school. Because he's sick, and because of COVID. COVID has turned our world upside down in a bad way. (Narla)

I noticed that it really started to really wear on him [teenage son] not being able to hang out with his friends, and he was starting to get sad… you could see the shift in the way that he was. (Sage)

Many of the mothers in our sample described their varied efforts to protect their children from the physical, social, and psychological impacts of the pandemic (i.e., minimizing media in the home; highly creative practices to keep children entertained, active and socially connected). In so doing, however, this was another layer of the emotional labor that mothers were performing during and after lockdown periods, and evidence of the intensification of ‘good' mother pressures and expectations during the pandemic.

#### Mothers' wellbeing and mental health

A few mothers described enjoying lockdowns, this was particularly the case for those mothers with older children (not babies or toddlers), without stressful jobs, some financial security and family support (i.e., sharing of childcare and domestic labor with partners). However, the majority of the mothers in our sample experienced what Dean et al. (2022) refer to as the mental over*load* of emotional labor (i.e., worrying about children's health and wellbeing; managing multiple roles and responsibilities within the home, wider families, work). Mirroring international research, the mothers in our study engaged in intensive forms of emotional labor through the daily management of their own and others' emotions (i.e., children, partner, parents, friends, workmates), but the chronic nature of this mental overload was “exhausting, leading to increased anxiety, depression and poorer physical health” for many (Dean et al., 2022, p. 19).

Across each of the groups (pregnant women, mothers with newborns, single and low-income mothers, and working mothers) some experienced severe mental health (i.e., depression) and “suicidal thoughts” (Narla) requiring medical treatment. For these women, the lack of access to social and medical support compounded their mental health issues:

Over time, my mental health just went so low, to a point where I told my social worker… I need help because I am not in a good headspace right now. I want to get onto things now before it gets worse, before I hurt myself or my kids. (Mila)

I was mentally unstable. COVID was the main trigger. I realized after that three-month lockdown, my anger toward the boys, I had been more snappy at them to a point where CYFs [Child, Youth and Family] had been called on me. I can't enjoy the boys as much as I would love to, because right now it's more like a burden. My therapist was going to send me to respite, that's how bad I am with my mental health at the moment… but I said there is absolutely no way I can go to respite… because I won't get my kids back again. (Narla)

I think typically mums put everyone else before themselves, and it is a huge mental load. Just that building up of stress, not getting enough sleep, exhaustion… I was getting resentful of having to do everything. I had panic attacks. Depression. I needed help, but I couldn't get hold of my psychotherapist. I turned up at [GP center], but they turned me away. They said, “We're only seeing urgent patients.” I was getting to that point where I was having harmful thoughts. I ended up calling Helpline. I just wanted to sit in my wardrobe. (Beth)

For many mothers in our study, the pandemic surfaced new affective relations with motherhood (i.e., anger, sadness, loss of enjoyment, resentment) that were contradictory to dominant discourses and social norms of ‘good motherhood' (Schmidt et al., 2023). While Mila, Narla and Beth each had difficulty accessing mental health support, it was only the Māori-Pacific mother (Narla) whose difficulties led to surveillance by the State (CYFs), with the ever-present threat of having her children removed from her care. As Stanley and de Froideville (2020) explain, discursive and institutional arrangements, and political and policy priorities, toward families in Aotearoa have long emphasized racialized risk and vulnerability, thus “re-emphasis[ing] colonial practices of viewing Māori children and young people as deficit-laden risks to be managed”, thus rendering Māori families as “perilously entrenched in welfare and justice systems” (526). In this context, not only did Māori mothers find it more challenging to access mental health support, but previous experiences of racial discrimination within the health and social services negatively impacted their willingness to express vulnerabilities and seek help.

Furthermore, our findings align with international research that has shown the heightened negative affect and mental distress among mothers during the pandemic was particularly heightened among those with babies, toddlers, and young children (Racine et al., 2021). Furthermore, the mothers in our sample without social and emotional support at home (and in their wider networks), and in economically difficult situations (i.e., single and low-income or welfare; income changes due to pandemic job loss in the family), described experiencing the most severe mental health impacts. The intersectional experiences of mothers on the margins must not be overlooked, particularly by those developing policies, practices, and support structures for families during periods of heightened stress, fear, and uncertainty.

#### Strategies of support

International research has identified a clear relationship between mothers' mental health and children's experiences of depression during the pandemic, thus highlighting the need for public health services that “support parents–and particularly mothers–in reducing individual stress and parenting stress” (Babore et al., 2023, p. 134). During the COVID-19 pandemic, however, such public health services were largely unavailable to most mothers in Aotearoa New Zealand. In this context, many mothers developed their own strategies to try to improve their mental health and wellbeing, and to support others. For example, mothers of young children described the importance of leaving the house for short periods of time:

The thing I do for my wellbeing is I actually take time out, even if it's just 10 min… I just tell the boys, “Look, I'm going to take 5–10 min, you guys sit down, watch TV and I'm just going to focus on me” (Mila)

The highlight of my week was going to the supermarket. I'd go on my own and it was great, just to get out of the house (Beth)

Other mothers engaged in physical activity or fitness (i.e., yoga, going for a walk, a home workout) as forms of escapism and stress relief. Some mothers described finding value in home-based beauty practices as a form of self-care: “I love my nails… that's my thing, my one guilty pleasure” (Lady). Others found themselves using alcohol, vaping, and caffeine as coping strategies during emotional challenging times:

There were times where I didn't have time to sit down and have a proper meal so coffee would be my go-to. Having coffee and a biscuit was my happy thing, comfort thing. …When I'm really, really angry at the boys, I would step outside, take 5 min and have a quick cuppa and vape. (Mila)

Some mothers found solace in other practices, such as online shopping. As Shelley explains: “I do a lot of online shopping where I put things in my cart and then never actually buy them. I find it therapeutic, it's a bit of escapism, it's time that I can take for me”. As Ncube et al. (2022) revealed in their study of young Black women in the US, there is a politics of self-care in such ‘self-soothing practices' as strategies to support themselves when systems of (formal and informal) support were inadequate.

According to the mothers in our sample, however, the most valued form of support for their wellbeing was the (partial and then full) return of childcare (Obeng et al., 2022):

Then [daughter] went back in [to childcare] Level 3, and my wellbeing, I was okay. It was stressful managing two children and not getting much sleep with an eight-month-old baby. I was quite glad that [daughter] could go to daycare. It helped so much. She has a lot of energy; she was driving me crazy! (Beth)

As soon as I closed that door [on the daycare van] and they took off, I celebrated. Now I'm able to do things I need to do for me. I clean things that I can't clean when the boys are around. Wiping down walls and grubby finger marks, and taking the dog for a walk (Mila)

While the importance of daycare and in-person schooling for mothers' labor force participation are well established (Collins, 2019; Ruppanner, 2020), our research highlights the critical role of childcare–inside and outside of the home–for mothers' (particularly single, low-income, and working mothers) mental health and wellbeing during and beyond the pandemic.

Across our sample, mothers described how the loss of formal and informal support networks impacted their ability to manage their multiple roles and responsibilities. For example, Beth felt like a “mum in a box”, and recognized that other mothers may be similarly struggling:

I think the pandemic has added that extra layer of stress and worry, because you lost your village… you're on your own. You're a mum and in a box with children, you haven't got the support, haven't got the help, haven't got the community.

Recognizing the challenges of other mothers, some of the women in our sample reached out to other families to offer support, even when they themselves were struggling:

It's so stressful, just trying to keep yourself together. You're trying to run a household and parent kids and be a teacher and work and yeah, be a wife and be there for yourself. And a friend to other people, because there's so many other people that are going through the same thing, so it's important to be there for them too. (Shelley)

Yeah, we did have a big bubble, I'm not going to lie. The other household was another single mum with three kids. She needed that as much as I did. (Mila)

Continuing, Mila explained her ongoing efforts to find support elsewhere (i.e., social media networks) when she needed empathy and connection with others who understood the challenges of being a single mother:

I'm on a lot of solo parent social media pages now on Facebook and Instagram and TikTok, so just seeing other solo parents going through the same thing and complaining about similar things made me feel less shit of a mum.

For those mothers unable to access the support they needed from their families, social services or health organizations, in-person and online networks with mothers in similar circumstances offered access to much-needed empathy, care, and compassion.

Through such informal networks, mothers supported one another, thus “cultivating communities of care” (Manzo and Minello, 2020). However, as Pri acknowledged, mothers of color often carry additional responsibilities in the home and community, and thus informal systems of support played a particularly important role in supporting friends and family: “The pressures on women and women of color during a pandemic are triple fold… checking in on all of my sisters, my friends, was important”. Here our findings align with research by Black feminist scholars in the US who, drawing upon Collins' (2000) concept of ‘othermothering', describe the ‘role overload' among Black women during the pandemic, particularly as they are “increasingly called upon to care for families and whole communities without compensation and support” (Laster Pirtle and Wright, 2021, p. 172). As a feminist intersectional lens on pandemic motherhood in Aotearoa highlights, the challenges facing mothers, and the strategies used to manage the many emotional, financial, and social stressors, vary widely. Thus, the systems of social, emotional, and medical support during times of great social disruption must acknowledge the intensified challenges for mothers, but also how multiple systems of oppression amplify the effects on some groups of mothers. While many mothers reach out to support other mothers, the double burden of care takes an emotional toll.

## Conclusion

This paper builds upon and extends research on pre- and pandemic mothering, highlighting the multiplicities of mothers' experiences during a global health emergency. Drawing upon interviews with 24 mothers living in Aotearoa during the COVID-19 pandemic, and engaging a feminist intersectional lens, we reveal how mothers' experiences during the pandemic varied considerably due to an array of intersecting factors (i.e., family and social support, economic stability, work responsibilities). As well as differences between pregnant women and mothers of newborn babies, single and low-income, and working mothers, we also highlight three common themes across our sample of mothers. All mothers were concerned for the health and wellbeing of their children, and went to great lengths to protect them from physical and socio-psychological harms during this period of great uncertainty, risk, stress and disruption. Their own wellbeing was also impacted in a range of ways and to varying extents due to the increased emotional overload they were carrying during lockdowns. Also, with formal and informal social support structures under considerable strain, many mothers engaged in additional emotional labor by reaching out, connecting with, and supporting other mothers. For all mothers, however, it was the return of childcare that offered the biggest relief to their health and wellbeing. Our analysis also revealed the lack of intersectionality in service provision, and limited understandings of what care entails (not just childcare, but also kinship and friendship), as well as mental health vulnerabilities created and/or exacerbated by the social isolation of pandemic lockdowns.

Yet despite some similarities, the pandemic experiences of Māori, Pacific, migrant and single mothers were further intensified by various forms of isolation, judgement, and discrimination. While previous research on pandemic motherhood has adopted intersectional approaches to examine the experiences of a group of mothers (i.e., single mothers, Black mothers), our study focused on a diverse sample of New Zealand mothers. In so doing, our research highlights the importance of intersectional approaches that recognize the highly variable impacts of such social disruption on mothers, and the need for targeted systems of support and care that prioritize the plurality of voices and experiences of mothers, and the urgency in developing culturally and gender responsive approaches that support the most vulnerable mothers during times of emergency. While pandemic lockdowns may seem to be a phenomenon from the past, this paper highlights the importance of an intersectional feminist lens for understanding and responding to the multiplicities of mothers' experiences, and their varied needs, during periods of radical social disruption and crisis. Importantly, if the intersectional needs of mothers are not adequately acknowledged and addressed, the risk is that pre-existing inequities are exacerbated for mothers and their children long after the event.

## Data availability statement

The datasets presented in this article are not readily available because the data is highly personalized and cannot be shared beyond the analyzed and anonymized data presented in the article. Requests to access the datasets should be directed to holly.thorpe@waikato.ac.nz.

## Ethics statement

The study involving humans was approved by University of Waikato Human Research Health Ethics Committee. The study was conducted in accordance with the local legislation and institutional requirements. The participants provided their written informed consent for participation in the study and for the publication of any potentially/indirectly identifying information included in the article. A “partial disclosure” option was approved by the Ethics Committee—the participants were assured that their names would not be disclosed, but consented to the publication of other descriptive aspects.

## Author contributions

HT: Conceptualization, Data curation, Formal analysis, Funding acquisition, Methodology, Project administration, Writing—original draft, Writing—review & editing. NB: Writing—review & editing. MN: Data curation, Writing—review & editing. GO'L: Data curation, Writing—review & editing. NA: Writing—review & editing.
